# Patients’ and informal carers’ insights into influences on prescribing in borderline personality disorder: a qualitative interview study in the UK

**DOI:** 10.1136/bmjopen-2025-108927

**Published:** 2025-12-03

**Authors:** Joshua Confue, Ian Maidment, Matthew Jones

**Affiliations:** 1 University of Bath, Bath, UK; 2 Lincolnshire Partnership Trust, Lincoln, UK; 3 Pharmacy, Aston University, Birmingham, UK

**Keywords:** Personality disorders, PSYCHIATRY, Prescriptions

## Abstract

**Abstract:**

**Objectives:**

To explore the patient and informal-carer reported factors that influence prescribing decisions in the management of borderline personality disorder (BPD).

**Design:**

The study employed a qualitative methodology of semi-structured interviews with patients and informal carers to examine perspectives on prescribing decisions and the factors shaping them.

**Setting:**

Interviews were conducted across both primary and secondary care settings in England.

**Participants:**

A total of 10 participants were recruited for the study, comprising eight females and two males, all aged 18 years or older. Participants either had a formal diagnosis of BPD or were informal carers of individuals diagnosed with BPD. All participants had experience with the prescribing of medication for the management of BPD.

**Results:**

Thematic analysis, employing both inductive and deductive strategies and informed by agency theory, yielded three interrelated themes: prescribing for symptom relief, the impact of risk on prescribing and difficulties in accessing services. Participants described medication as a necessary means of managing distress, especially when access to psychological therapies was constrained. Despite awareness of potential adverse effects, many expressed a strong desire to make their own decision around medication.

**Conclusions:**

Improving clarity around the likelihood of both symptomatic relief and potential adverse effects through co-designed informational resources may support more informed decision-making in the treatment of BPD. Furthermore, to change prescribing patterns, systemic gaps in the provision of long-term psychological therapies must be addressed.

**STRENGTHS AND LIMITATIONS OF THIS STUDY**

- This study recruited across multiple avenues, including five NHS Trusts, covering both primary and secondary care settings.
- This study did not examine prescribing influences from healthcare professionals’ perspectives, although this has been investigated separately.

## Introduction

 Borderline personality disorder (BPD) is a mental health condition which presents with intense fears of abandonment and difficulty regulating one’s emotions.^1^ Patients with BPD are recognised as a cohort with some of the highest unmet needs^1^ and a review of cases in Denmark between 1995 and 2011 identified a roughly four times higher mortality rate in patients with BPD than in the general population.^2^

Estimates of the lifetime prevalence of BPD vary across studies, ranging from approximately 0.7% to 2.7% in the general population.^3 4^ This prevalence, combined with the severity of the condition, may explain the significant proportion of patients with BPD in mental health services, with patients with BPD constituting 15–28% of service users.^5^

Although the prescribing of medication is the predominant form of treatment for a range of psychiatric illnesses,^6^ no medications are licensed for the treatment of BPD since there is currently limited evidence of efficacy.^7 8^ Instead, the primary recommended treatment is psychological therapy.^7 8^ Despite this, the frequency of prescribing in this cohort is high. For example, Bridler *et al* estimated from a 10-year European retrospective study that over 70% of patients with BPD were prescribed one or more psychotropic medications.^9^ Commonly prescribed medications include antidepressants, mood stabilisers, antipsychotics and anxiolytics, which are often administered over extended periods of time.^8^

This raises the question of what is driving the disparity between guidelines and real-world practice. Modern prescribing is ideally based around the shared decision-making process, with the decision to prescribe made jointly between the prescriber and the patient or their representative. A 2025 systematic review on influences on prescribing in BPD identified several factors associated with the prescribing process, in particular the influence of the patient-healthcare professional (HCP) relationship and the available care pathways on prescribing.^10^ Notably, despite international advocacy for patient-centred care and active patient involvement in prescribing decisions, only two papers were identified that involved patients and none that involved informal carers. Across all the qualitative studies identified in this review, only 27 patients were interviewed, 20 of whom were recruited to a study focused only on the use of clozapine.^10^

These two studies were conducted in notably different environments of a forensic inpatient and a community outpatient service.^11 12^ However, both highlight the role of medication in the care of individuals with BPD. Dickens found that, despite acknowledgement of the risks, participants generally had a positive view of pharmacological treatment (clozapine), citing reports of positive cognitive and affective changes reported by patients.^11^ Contrastingly, Patel reported that patients often expressed uncertainty about the effects of medication, while paradoxically also frequently describing its powerful impact.^12^ Overall, the authors focused on the importance of interpersonal relationships and the role of prescribing in facilitating these experiences.

Given the limited efficacy of medications in BPD and the high rates of prescribing, there is a need to better understand the factors driving these practices. This study seeks to expand the limited existing evidence base by exploring the influences shaping prescribing decisions from both patient and carer perspectives to better inform our understanding of the factors behind prescribing in BPD.

## Materials and methods

### Study design

Qualitative semi-structured interviews were used to explore the perspectives of both patients and informal carers. Informal carers are defined as a family member, partner or friend providing unpaid support.

The research team comprised three pharmacists: JC (male, doctoral candidate), a clinical service manager and doctoral student; IM (male, PhD), a Professor of Clinical Pharmacy; and MJ (male, PhD), a Senior Lecturer. All three researchers had received training in qualitative research methodologies, with IM and MJ possessing prior experience in applying these methods. The team had a shared research interest in the study topic and had recently co-authored a systematic review on the subject.^10^ Four participants involved in treating and living with BPD helped design the study. Recruited via interest groups, they provided feedback through individual consultations. Their input supported the development of the protocol and related materials, particularly by highlighting the importance of relational factors in decision-making.

Due to the limited research available on factors influencing prescribing in BPD, the research team chose Agency Theory as the foundational theoretical model for this study.^13^ It was selected as a theoretical basis that encompasses relational and systemic factors that shape decision-making in prescribing, while making minimal assumptions about influences.

Agency Theory suggests that an ‘agency relationship’ occurs when principals (patients) grant authority to agents (prescribers) to act on their behalf.^14^ Within the context of prescribing, this implies that the choice of medications for treating health conditions represents an agency relationship.^14^ This model also supports the notion that the patient-HCP relationship is crucial to the process, which is a consistent conclusion found in the existing literature. In the early phases of the study, Agency Theory was utilised to outline the core relationship in prescribing.

### Patient or public contribution

While the research team did not contain members of the public or patients, there was engagement with the public and individuals with lived experience of BPD in the formation of the project plan and proposal. These discussions highlighted the importance of medication and prescribing in their journey, as well as the significance of patients’ desires and understanding in the prescribing process.

Four people involved in treating, caring for and living with BPD participated in the design of this study. They were recruited through special interest groups and took part in individual consultations. They supported the importance of the research topic and the proposed methods. Their feedback was used to refine the protocol and supporting materials (including the interview schedule) by simplifying the language, most notably by simplifying questions and acknowledging power dynamics in the decision-making process.

### Participants, recruitment and sampling

Participants were eligible to participate if they were aged 18 years and older and had been prescribed medication for BPD or cared for someone who had. Patients were unable to participate if they were treated outside of England, Wales and Scotland. Participants were excluded if they, the research team, or their community psychiatric nurse or responsible clinician (for those referred by HCPs) believed that participation posed a significant risk of harm.

Recruitment was guided by the principle of information power, with an initial recruitment target of up to 20 participants being set. This estimate was based on existing qualitative research and our prior estimation of the sample size required to generate adequate insight. Yet, the final sample was determined by the richness and sufficiency of the data to address the study aims.^15^

Participants were recruited through five NHS Mental Health Trusts, with additional promotion via charitable organisations (such as community centres) and ‘snowballing’ via previous participants.^15^ Recruitment activities included the dissemination of information via posters, through email communications, as well as during relevant meetings and educational sessions conducted by participating NHS Trusts. To mitigate the risk of imposter participants, recruitment was not conducted through social media. All participants voluntarily contacted the research team to express their interest in taking part. Prior to their interview, all potential participants were given a participant information sheet explaining the study’s background, aims and procedures, after which written consent was obtained prior to conducting all interviews. All participants were offered a £30 voucher for their participation.

### Data collection and analysis

All interviews were carried out by JC in private locations or online, documented through digital recordings, and later transcribed by the interviewer (see COREQ checklist for further details in online supplemental appendix A).

A new interview topic guide was developed (refer to online supplemental appendix B), with questions informed by Agency Theory, consultations with HCPs and patients, and insights derived from a systematic review of the existing literature.^10^ The development process was conducted in collaboration with academic supervisors (IM and MJ). The topic guide was designed to explore three core assumptions of Agency Theory: the presence of incomplete information for both the principal and the agent, the divergence in their goals and the difference in their appetite for risk. To investigate these assumptions, the interview guide included questions regarding medication options, treatment duration, effectiveness, evaluation of treatment responses, educational resources and self-identified factors influencing prescribing decisions.

Following transcription, the anonymised text was uploaded into Nvivo 11 for analysis. The research team utilised an inductive and deductive thematic analysis approach, adhering to the steps outlined by Braun and Clarke (2006),^16^ which involved familiarisation with the data, code development, theme identification, theme review, theme definition and naming, and the final reporting of themes.

After initial coding of the first three transcripts by JC, the research team met to review the preliminary coding and provide feedback. Once the initial codes were established and agreed on, the team engaged in ongoing discussions to debate and refine potential themes arising from these codes. After naming the themes, the research team reconvened to evaluate and analyse the identified themes using a mind map to facilitate discussion. This iterative process ensured comprehensive analysis, refinement and alignment before final reporting of themes.

**Table 1:** Demographics of participants

No	Role	Recruitment setting	Age (years)	Ethnicity	Gender
001	Patient	Primary care	18–21	White British	Female
002	Patient	Primary care	21–30	White British	Female
003	Patient	Primary care	21–30	White British	Male
004	Patient	Primary care	21–30	White British	Female
005	Carer	Secondary care	31–40	White British	Male
006	Carer	Secondary care	50–59	White British	Female
007	Patient	Primary care	21–30	Black British	Female
008	Patient	Primary care	21–30	White British	Female
009	Patient	Primary care	21–30	White British	Female
010	Patient	Primary care	21–30	White British	Female

## Results

**Figure 1:**
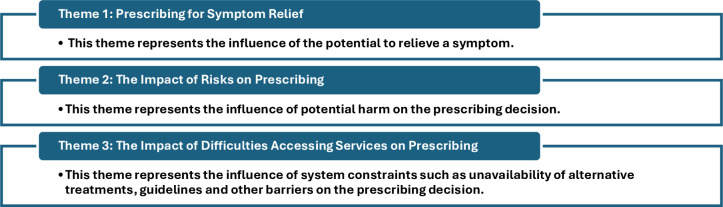
Description of three main themes generated.

10 interviews were completed with a range of patients and informal carers (table 1). After securing written, informed consent, the interviews had a mean length of 33 min (range: 16–56 min). Analysis of the transcriptions led to the creation of three main themes describing influences on the decisions of prescribing in BPD (figure 1).

### Prescribing for symptom relief

Participants of this study reported that medication was a significant part of their treatment and reflected that, in certain cases, it could relieve the symptoms of BPD:

I think medication has really helped me, Participant-008 (Patient)

[a] lot of the symptoms became really, really severe for her. Like I said, it [medication] helps with some of them… Participant-005 (Carer)

However, not all participants expressed that medication could help them:

Medications never really worked for me for anything like that, so […] I'm not sure, like, looking back at it now, with different choices made. Participant-004 (Patient)

Moreover, participants reported several more specific perceived benefits associated with pharmacological interventions, in particular that they played a meaningful role in the overall management of their mental health:

We did find some medication did reduce the intensity of the some of the more, I guess, erratic, explosive behaviour that came with my presentation. Participant-005 (Carer)

While the identified class of medication patients found to be beneficial was inconsistent, the positive impacts reported included the dulling of intense emotions and the provision of temporary relief, both of which served to facilitate engagement in daily and therapeutic activities, thus supporting broader recovery efforts:

[Medication] doesn't stop the emotion, but it does blunt them slightly. So it’s easier for me to live my life basically, and I think it would find it very hard to go through therapy in that without that Participant-008 (Patient)

Sometimes, I think you need the medication to be able to do the therapy, to be perfectly honest. Participant-009 (Patient)

Medications were also credited with reducing the severity and frequency of emotional outbursts, contributing to the ability to keep oneself safe:

It stops some of the major instability at that time. Participant-009 (Patient)

[Medications] have helped to keep me safe, somewhat Participant-001 (Patient)

When exploring what influenced participants’ choices around medication, participants placed great weight on their own research:

[I: What influenced your choice around medication?] It was just internet searches really… Participant-008 (Patient)

Many participants reported using internet forums and social media (such as TikTok) as part of their own research, in particular on the reports of others with lived experience of BPD:

Mainly websites and online forums, those kinds of thing. I, I've looked online for some general information around medication[s], how they work, the side effects, but the most helpful is definitely […] places like Reddit, or other forums Participant-007 (Patient)

a big thing to me, might be, how it’s affected other people like me, […] have other people reported that it’s helped them, it’s improved their mood, it’s helped them managing it, live their lives, manage their emotions, I think that’s really what’s important to me” Participant-010 (Patient)

Among participants who described wanting to start or stop medication, there seemed to be a greater belief in self-identified resources, internet forums and social media, rather than the information provided by HCPs.

I don't think and I [don’t] think the material from hospitals would influenced me a lot Participant-010 (Patient)

### The impact of risks on prescribing

An awareness of the risks associated with medication was also reported as an important influence on prescribing. However, there was significant variation in what risks were prioritised by patients, with participants expressing that they knew about some of the risks and benefits and NICE guidance through their own research, yet they had arrived at their decision in spite of this knowledge:

I feel the benefit outweighs the risk of side effects. Participant-001(Patient)

[I: But you weren't concerned around the side effects, the olanzapine?] Yeah, it’s not perfect, but I feel like my moods aren’t swinging as drastically. Participant-007 (Patient)

Participants often highlighted the side effects of medication, as well as potential interactions, as a significant factor in their decision making:

[…] all the side effects or individual, but they're all side effects that would have been catastrophic Participant-005 (Carer)

They make you fat, they make you sleepy, they turn you off on the difficulties stuff, but they also cut you off from the nice stuff, and this is [a] constant balance. Participant-009 (Patient)

A number of participants expressed that when deciding about switching medications, fear around destabilisation of their mental state or other adverse effects was a significant factor:

[I: What would make you decide against or for a medication?] For me, it would be side effects, […] in other people who are similar to me Participant-001 (Patient)

Participants also expressed that they felt medication was prescribed on occasion due to the HPCs’ perception of their (the patient’s) risk, such as self-harming. They felt that HCPs started them on medication because they were perceived as ‘risky’”:

I honestly think it’s a bit of panic, like. But, I presented to my doctors or to services, whatever that might be, [as] a bit of pain in people and I think people wanted a little bit of a ‘we've done this, so this is OK’ Participant-004 (Patient)

This may reflect elements of defensive practice on the part of HCPs as they attempt to mitigate liability. It may also be an expression of countertransference and HCPs’ desires to manage their own uncomfortable emotions, with the act of prescribing medication aiming to manage HCPs’ sense of helplessness around the risks presenting in patients, or other negative emotions.^17^

### The impact of difficulties accessing services on prescribing

Participants repeatedly spoke about the challenges they experienced in accessing care, raising concerns regarding access to support at both the primary and secondary care levels, reporting that long delays or gaps in support led to pharmacological treatment being initiated as a more immediate intervention:

… you know you're on a waiting list, there’s nothing we can do right now, the only alternative we can offer you at the moment is medication Participant-002 (Patient)

The following description outlines the range of difficulties participants identified in accessing services that may influence prescribing for those with BPD, which arose from multiple causes. Significant emphasis was placed on a perceived lack of understanding, coupled with negative inferences from HCPs:

It was huge amounts of judgment… I needed to go to A&E, [and] then those professionals, some were kind of like OK, just did their job, but others were incredibly judgmental. Participant-004 (Patient)

This sentiment of stigma was coupled by many participants with services being unhelpful, citing limited therapeutic value, being dismissed and a lack of individualised support:

You got acute ward with very unwell patients with lots of diagnoses and [and] the more there’s also a real stigma around [BPD]… So, it’s: lack of how to deal with it [BPD], and understanding, and how to approach the person, you know, instead of [dealing] with real ‘problem person’ Participant-006 (Carer)

Even where services were seen positively, a consistent issue was the slow pace of, or lack of access to, a particular type of treatment (often psychological therapy):

I was put on a waiting list for adult mental health services for a year and a half. I wasn't seen by anybody Participant-002 (Patient)

My personal experience: there’s no difference with depressive, depressive lows. We get anxiety; we get all of those broad spectrum of emotions, very intensely and so why that help isn't available Participant-002 (Patient)

Additionally, there was reported frustration with rigid, criteria-led approaches to service eligibility, highlighting systemic barriers within mental health services that limit access to care and complicate the care pathway:

[mental health services] a lot of them have criteria, and I haven’t met their criteria Participant-001 (Patient)

Some of these reported barriers in accessing services were compounded by diagnostic uncertainty or changing diagnoses:

Yes, so, they told me like I had depression, and then it wasn't depression. And then, like, it was me, and then it was like it could be this, it could be that. You know, most people I saw have had and different opinion. It wasn't good. It wasn't great. It wasn't very helpful Participant-003 (Patient)

Moreover, the time-limited nature of support was seen as insufficient by participants (often reported to be restricted to only a few weeks). With individuals feeling returned to the start of their journey through the care pathway, without meaningful progress being made in their condition:

…they only want to support you for a few weeks Participant-001 (Patient)

So, no. For me, like, services weren't right, […] never really helped me, they always kind of were dismissive Participant-003 (Patient)

These challenges or complete inability to access certain services seem to culminate in instances where participants engage with health services, and the only path on offer is medication:

We went to access services… [they said] we can't help you, so you sort out the BPD… there was no goal [if] she didn't want medication Participant-005 (Carer)

## Discussion

This study generated three themes describing influences on the prescribing process in BPD, shaping decisions and outcomes for patients. Themes 1 and 2 described the potential impact of medication on patients and the influence of patients’ and HCPs’ beliefs surrounding treatment and adverse effects on the prescribing process. Theme 3 described the effect that difficulties accessing services had on the prescribing process. These themes were evident across the accounts of both patients and informal carers. While carers provided additional insights, particularly in relation to risk, monitoring and support needs, the perspectives they offered were largely consistent with those expressed by patients. Our findings suggest that prescribing, at least in part, reflects an effort to obtain symptomatic relief where access to alternative treatment is limited or difficult. Participants appear to weigh the potential adverse effects of medication against the risks associated with leaving their condition untreated.

Prescribing is a complex task, especially in BPD, given the limited evidence base.^8^ It can be impacted by a range of influences, but to date, there has been limited research on factors affecting prescribing in this population.^10^ Although this study was relatively small, it constitutes a significant increase in the body of evidence. Prior to this, we identified only two previous studies, with a total of 27 participants, that explored patient perspectives of influences on prescribing in BPD, and one of these focused solely on clozapine. In addition, as far as we are aware, this is the first study that has included the opinion of informal carers.

Recruitment for the study took place across five NHS Trusts and included patients from both primary and secondary care settings, providing a more representative sample of the UK population than previous studies. The primary limitation of this study is the small sample size. Recruitment was stopped after the completion of 10 interviews, as all available advertising avenues had been exhausted and no additional interest was observed over a 1-month period. Although this study was relatively small, the data collected was of sufficient quality and relevance to address the study aims and to make a meaningful contribution to the field. This sample size was partly due to the limited trust between patients and HCPs.^18^ This lack of trust may have been reinforced by the requirement for the researchers to have contact details for the HCP or support network of each participant. Second, the study did not explore prescribing influences from the perspectives of HCPs, although this aspect has been investigated through a separate study (paper in preparation).

### Prescribing for symptom relief

Participants’ belief in the potential benefits of medication emerged as a key driver of prescribing, shaping practices and clinical decisions. The idea that patients believe medication can be of benefit is present in the existing literature. Patel and Konstantinidou^12^ found that patients with BPD reported medication as having a profound effect on both the mind and body, and Dickens^11^ reported on patients’ beliefs around the positive effects of medication, though the nature of these effects was not always clearly defined by participants. There are several potential explanations for the tension between patient experience and evidence-based practice, including the placebo effect^19^ and the sense of validation associated with receiving a prescription^20^ emerging as the most prominent. Both phenomena are recognised as being influenced by the quality of interpersonal interactions,^21^ highlighting the crucial role of HCPs in supporting individuals to make informed decisions about their medications.

Patients’ expectations, and therefore placebo responses, are often shaped by the sources of information utilised. Many participants placed greater weight on the lived experiences of others with the same condition than on HCPs. The use of social media was particularly prominent, not only reflecting its widespread use in society, but perhaps a particular appeal in this cohort. A 2024 study found that this community may be a more appealing source of information, with social media offering a distraction from interpersonal problems, a reassurance that people are still cared for, and an outlet for those struggling with self-esteem or confidence.^22^ While such sources can provide valuable support, they are also very variable and often anecdotal. This raises important questions about the extent to which patients can be regarded as informed decision-makers and about the influence of preconceived expectations on treatment outcomes.

With regard to the validating effect of medication, the act of prescribing can provide benefits beyond the pharmacological; it can provide secondary benefits and shift what might be seen as a personal failing to a recognised medical condition beyond an individual’s control.^23^ The use of prescribing as a means of validation and of establishing or maintaining a positive therapeutic relationship has previously been reported by HCPs as a driver of prescribing^10^ (Paper in preparation), due in part to the perceived benefits such relationships can provide.^24^ In contrast, the importance of the therapeutic relationship did not emerge as a prominent theme influencing prescribing within this study. Instead, discussions of the therapeutic relationship were more likely to surface when negative or strained relationships with HCPs were perceived as significant barriers to accessing care. The reasons for this difference in the perceived role of the patient-HCP relationship are unclear. It is possible that professional training, which emphasises the therapeutic relationship and the limited pharmacological evidence in BPD, may have influenced HCPs’ perceptions. Conversely, patients may have attributed the benefit from the therapeutic relationship primarily to the medication or other intervention, rather than to the relational aspects of care.^19^

### The impact of risks on prescribing

In this study, some participants felt that medication was needed to reduce the chance of ‘risky’ behaviours. This overlaps with Theme 1 and the idea that medication can provide relief and reduce these behaviours. This aligns with reports from earlier studies where medication was felt by patients to ‘slow things down’ and reduce these behaviours.^11^ Interestingly, a number of participants believed they were prescribed medication due to being perceived as high risk by the HCPs involved, even though they believed the medication was not helpful. These reports may indicate instances of ‘countertransference’, where the medication is being prescribed to manage the prescriber’s feelings of concern or helplessness.^17^ This is not to suggest that HCPs or patients overlooked the potential harms of medication. Instead, they indicate that they are balancing the perceived risk, including the risk of not treating, and the possible benefit of treatment options. Concerns around potential adverse effects, and even prior adverse effects experienced by participants from other medications, were also reported as influencing decisions around prescribing. These highlighted concerns reinforce documented factors in non-adherence with medication in general.^25^ Nevertheless, our findings suggest that the importance placed on side effects is different between those with BPD and HCPs, and that patients may, particularly when experiencing distress, assign less importance to the risk of side effects. This reflects the findings of a 2016 focus group in patients with BPD (on clozapine), which found that participants viewed side effects as relatively unimportant^11^ despite clozapine being considered to have a high side effect burden by HCPs.^26^ This may be because fear of side effects can differ based on personal and clinical attributes, as well as the quality of information individuals use, among other factors.^27^

### The impact of difficulties accessing services on prescribing

Individuals who experience BPD often report difficulties in accessing support from the healthcare system.^28^ The high levels of prescribing within this group may not solely reflect individual clinical decisions but also structural issues within the healthcare system. As a result, medication may be viewed as a pragmatic solution since alternative therapeutic options are often inaccessible. These reflect wider strains on mental health services. A Royal College of Psychiatry report indicates that almost a quarter of mental health patients wait over 12 weeks for treatment to start, with 12% waiting longer than 6 months and 6% over a year. During this time, 78% of these individuals resort to emergency services or a mental health crisis service to manage their mental health.^29^ This inability to access alternative therapies, whether genuine or perceived, leaves patients with only a single means of treatment: medication. This reflects a 2005 study examining prescribing in depression, which found that General Practitioners (GPs) felt compelled to prescribe when alternative treatments were unavailable or involved long waiting times.^30^

These systemic barriers may be further exacerbated by stigma among HCPs towards patients with BPD.^31 32^ Stigma is a proven barrier to engaging in care across various health conditions worldwide.^33^ In particular, there are indications that these misconceptions may negatively impact how patients navigate and progress through treatment pathways,^34^ creating another barrier to patients accessing specialist services or input. This, in turn, may drive them toward the prescribing of medication in primary care, which is often the initiation point of treatment pathways. Alternatively, even where alternative therapies can be accessed, if there are extended waiting lists, this may not be tolerable to patients in distress. Conversely, medication can be accessed almost immediately.

### Recommendations for further research

The beliefs of patients and carers, described in the results of this study, provide context and a deeper understanding of the influences on prescribing in BPD. Despite this, further work is needed to develop our insight into the perceptions of patients and informal carers. Future research should aim to include a larger and more diverse sample, particularly focusing on individuals from underrepresented demographic groups, including those from non-white backgrounds. Additionally, future studies may wish to consider variations in socioeconomic status in their sampling, given the suggested impact that difficulties in accessing services can have on the prescribing process.

In the context of BPD, ethics committees governing mental health research should look to facilitate meaningful patient engagement through the re-evaluation of eligibility criteria to promote equitable participation. One example of this was the current requirement for participants to divulge contact details for their HCPs, even for non-intervention studies, which can inadvertently infringe on participant autonomy and thus deter engagement. Through the elimination of such barriers, the voices of individuals with severe mental illnesses can be present in research, advancing justice, one of the four pillars of ethical research.^35^

In addition, at present, there is a lack of research exploring whether participation in non-intervention studies poses inherently greater risks for individuals with BPD than the general public. Addressing this gap could help resolve uncertainty and inconsistency in the decisions of ethics committees.

## Conclusion

This study indicates that, from the perspective of patients and carers, three dominant themes influence the use of medication in patients with BPD: the belief that medication provides symptomatic relief, risks around unmet needs and adverse medication effects and a lack of timely alternative options.

A number of participants reported seeking medication in an attempt to obtain symptomatic relief, though this was not universal, and as such, HCPs should not assume that the provision of medication is always what a patient desires. Participants expressed a desire to make their own risk–benefit decisions based on personal values and priorities. Many also perceived systemic barriers that hindered their ability to access what they considered to be appropriate care, in particular psychological therapies and other forms of specialist input, leaving medication as one of the few available options.

Improved clarity around the probability of symptomatic relief and adverse effects, through the development of co-designed information resources, could facilitate better informed care in BPD. Additionally, in order to make it possible for non-pharmacological choices to be accessible to both patients and HCPs, the lack of provision for long-term psychological services must be addressed.

## Supplementary material

10.1136/bmjopen-2025-108927 online supplemental file 1

10.1136/bmjopen-2025-108927 online supplemental file 2

## Data Availability

Data may be obtained from a third party and are not publicly available.

## References

[1] Wlodarczyk J, Lawn S, Powell K (2018). Exploring General Practitioners’ Views and Experiences of Providing Care to People with Borderline Personality Disorder in Primary Care: A Qualitative Study in Australia. Int J Environ Res Public Health.

[2] Kjær JN, Biskin R, Vestergaard CH (2015). A Nationwide Study of Mortality in Patients with Borderline Personality Disorder. Eur Psychiatry.

[3] Dahlenburg SC, Bartsch DR, Gilson KJ (2024). Global prevalence of borderline personality disorder and self-reported symptoms of adults in prison: A systematic review and meta-analysis. Int J Law Psychiatry.

[4] Leichsenring F, Fonagy P, Heim N (2024). Borderline personality disorder: a comprehensive review of diagnosis and clinical presentation, etiology, treatment, and current controversies. World Psychiatry.

[5] Gunderson JG, Herpertz SC, Skodol AE (2018). Borderline personality disorder. Nat Rev Dis Primers.

[6] Waszczuk MA, Zimmerman M, Ruggero C (2017). What do clinicians treat: Diagnoses or symptoms? The incremental validity of a symptom-based, dimensional characterization of emotional disorders in predicting medication prescription patterns. Compr Psychiatry.

[7] NICE (2009). Borderline personality disorder. Conditions and diseases.

[8] Stoffers-Winterling JM, Storebø OJ, Pereira Ribeiro J (2022). Pharmacological interventions for people with borderline personality disorder. Cochrane Database Syst Rev.

[9] Bridler R, Häberle A, Müller ST (2015). Psychopharmacological treatment of 2195 in-patients with borderline personality disorder: A comparison with other psychiatric disorders. Eur Neuropsychopharmacol.

[10] Confue J, Maidment I, Jones S (2025). Factors That Influence Prescribing in Borderline Personality Disorder: A Systematic Review. Personal Ment Health.

[11] Dickens GL, Frogley C, Mason F (2016). Experiences of women in secure care who have been prescribed clozapine for borderline personality disorder. Borderline Personal Disord Emot Dysregul.

[12] Patel D, Konstantinidou H (2020). Prescribing in personality disorder: patients’ perspectives on their encounters with GPs and psychiatrists. *Fam Med Community Health*.

[13] Hoti K, Hughes J, Sunderland B (2011). Pharmacy clients’ attitudes to expanded pharmacist prescribing and the role of agency theory on involved stakeholders. Int J Pharm Pract.

[14] Ali Murshid M, Mohaidin Z (2017). Models and theories of prescribing decisions: A review and suggested a new model. Pharm Pract (Granada).

[15] Malterud K, Siersma VD, Guassora AD (2016). Sample Size in Qualitative Interview Studies: Guided by Information Power. Qual Health Res.

[16] Braun V, Clarke V (2006). Using thematic analysis in psychology. Qual Res Psychol.

[17] Shapiro-Thompson R, Fineberg SK (2022). The State of Overmedication in Borderline Personality Disorder: Interpersonal and Structural Factors. Curr Treat Options Psychiatry.

[18] Maidment ID, Brown P, Calnan M (2011). An exploratory study of the role of trust in medication management within mental health services. Int J Clin Pharm.

[19] Colloca L (2018). Preface: The Fascinating Mechanisms and Implications of the Placebo Effect. Int Rev Neurobiol.

[20] Kinghorn WA, Nussbaum AM (2021). Prescribing together: a relational guide to psychopharmacology.

[21] Tavel ME (2014). The placebo effect: the good, the bad, and the ugly. Am J Med.

[22] Collins M, Grant JE (2024). Social media addiction and borderline personality disorder: a survey study. Front Psychiatry.

[23] Schnell T, Kehring A, Moritz S (2021). Patients responses to diagnoses of mental disorders: Development and validation of a reliable self-report measure. Int J Methods Psychiatr Res.

[24] Baier AL, Kline AC, Feeny NC (2020). Therapeutic alliance as a mediator of change: A systematic review and evaluation of research. Clin Psychol Rev.

[25] Laba TL, Essue B, Kimman M (2015). Understanding Patient Preferences in Medication Nonadherence: A Review of Stated Preference Data. Patient.

[26] Iqbal E, Govind R, Romero A (2020). The side effect profile of Clozapine in real world data of three large mental health hospitals. PLoS One.

[27] Smith LE, Webster RK, Rubin GJ (2020). A systematic review of factors associated with side-effect expectations from medical interventions. Health Expect.

[28] Klein P, Fairweather AK, Lawn S (2022). Structural stigma and its impact on healthcare for borderline personality disorder: a scoping review. Int J Ment Health Syst.

[29] Royal College of Psychiatrists (2022). Hidden waits force more than three quarters of mental health patients to seek help from emergency services. https://www.rcpsych.ac.uk/news-and-features/latest-news/detail/2022/10/10/hidden-waits-force-more-than-three-quarters-of-mental-health-patients-to-seek-help-from-emergency-services.

[30] Hyde J, Calnan M, Prior L (2005). A qualitative study exploring how GPs decide to prescribe antidepressants. Br J Gen Pract.

[31] Masland SR, Victor SE, Peters JR (2023). Destigmatizing Borderline Personality Disorder: A Call to Action for Psychological Science. Perspect Psychol Sci.

[32] Navarre KM (2025). “You sure she’s not making this up?”: A qualitative investigation of stigma toward adults with borderline personality disorder in physical healthcare settings. Personal Ment Health.

[33] Stangl AL, Earnshaw VA, Logie CH (2019). The Health Stigma and Discrimination Framework: a global, crosscutting framework to inform research, intervention development, and policy on health-related stigmas. BMC Med.

[34] Aviram RB, Brodsky BS, Stanley B (2006). Borderline personality disorder, stigma, and treatment implications. Harv Rev Psychiatry.

[35] Mallia P (2003). Biomedical ethics: The basic principles. BMJ.

