# Supplementary material for: Patients’ and informal carers’ insights into influences on prescribing in borderline personality disorder: a qualitative interview study in the UK
Source: BMJ Open. 2025 Dec 3;15(12):e108927. doi: 10.1136/bmjopen-2025-108927 (PMC12682173; doi:10.1136/bmjopen-2025-108927)
Supplement: online supplemental file 1 [file bmjopen-15-12-s001.docx]

Appendix-A Coreq Checklist

| Domain 1: Research Team & Reflexivity | | |
| --- | --- | --- |
| Personal characteristics | | |
| 1. Interviewer/facilitator | Which author(s) conducted the interview or focus group? | Pages 5 & 6 |
| 2. Credentials | What were the researcher’s credentials? (e.g. PhD, MD) | Page 4 |
| 3. Occupation | What was their occupation at the time of the study? | Page 4 |
| 4. Gender | Was the researcher male or female? | Page 4 |
| 5. Experience and training | What experience or training did the researcher have? | Page 4 |
| Relationship with participants | | |
| 6. Relationship established | Was a relationship established prior to study commencement? | Page 5 and 6 |
| 7. Participant knowledge of the interviewer | What did the participants know about the researcher? (e.g. personal goals, reasons for doing the research) | Page 4 , In addition participant information leaflets contained information on the researchers name, organisation, supervisor and purpose of the research |
| 8. Interviewer characteristics | What characteristics were reported about the interviewer/facilitator? (e.g. bias, assumptions, reasons and interests in the research topic) | Page 4 |
| Domain 2: Study Design | | |
| Theoretical framework | | |
| 9. Methodological orientation and theory | What methodological orientation was stated to underpin the study? (e.g. grounded theory, discourse analysis, ethnography, phenomenology, content analysis) | Pages 5 & 6 |
| Participant selection | | |
| 10. Sampling | How were participants selected? (e.g. purposive, convenience, consecutive, snowball) | Pages 5 & 6 |
| 11. Method of approach | How were participants approached? (e.g. face to face, telephone, mail, e-mail) | Pages 5 & 6 |
| 12. Sample size | How many participants were in the study? | Table 1 |
| 13. Non-participation | How many people refused to participate or dropped out? Reasons? | NA – Participants Contacted the Research Team |
| Setting | | |
| 14. Setting of data collection | Where was the data collected? (e.g. home, clinic, workplace) | Pages 5 & 6 |
| 15. Presence of non-participants | Was anyone else present besides the participants and researchers? | NA- No one outside of participants and researcher was present |
| 16. Description of sample | What are the important characteristics of the sample? (e.g. demographic data, date) | Table 1 |
| Data collection | | |
| 17. Interview guide | Were questions, prompts, guides provided by the authors? Was it pilot tested? | Pages 5 & 6 |
| 18. Repeat interviews | Were repeat interviews carried out? If yes, how many? | NA – No repeat interviews were conducted |
| 19. Audio/visual recording | Did the research use audio or visual recording to collect the data? | Pages 5 & 6 |
| 20. Field notes | Were field notes made during and/or after the interview or focus group? | Pages 5 & 6 |
| 21. Duration | What was the duration of the interviews or focus group? | Pages 5 & 6 |
| 22. Data saturation | Was data saturation discussed? | NA- Data saturation was not utilised , instead the concept of information power was utilised.  This is detailed on Page 8 |
| 23. Transcripts returned | Were transcripts returned to participants for comment and/or correction? | NA- Transcript were not returned to participants |
| Domain 3: Analysis & Findings | | |
| Data analysis | | |
| 24. Number of data coders | How many data coders coded the data? | Page 6 |
| 25. Description of the coding tree | Did authors provide a description of the coding tree? | NA- No |
| 26. Derivation of themes | Were themes identified in advance or derived from the data? | Derived from Data |
| 27. Software | What software, if applicable, was used to manage the data? | Pages 5 |
| 28. Participant checking | Did participants provide feedback on the findings? | NA- No |
| Reporting | | |
| 29. Quotations presented | Were participant quotations presented to illustrate the themes/findings? Was each quotation identified? (e.g. participant number) | Yes |
| 30. Data and findings consistent | Was there consistency between the data presented and the findings? | Yes  Figure 1 (Yes)  Yes |
| 31. Clarity of major themes | Were major themes clearly presented in the findings? |  |
| 32. Clarity of minor themes | Is there a description of diverse cases or discussion of minor themes? |  |

Developed from: Tong A, Sainsbury P, Craig J. Consolidated criteria for reporting qualitative research (COREQ): a 32-item checklist for interviews and focus groups. *International Journal for Quality in Health Care*. 2007. Volume 19, Number 6: pp. 349 – 357
