# Supplementary material for: Patients’ and informal carers’ insights into influences on prescribing in borderline personality disorder: a qualitative interview study in the UK
Source: BMJ Open. 2025 Dec 3;15(12):e108927. doi: 10.1136/bmjopen-2025-108927 (PMC12682173; doi:10.1136/bmjopen-2025-108927)
Supplement: online supplemental file 2 [file bmjopen-15-12-s002.docx]

Appendix B

Interview Topic Guide

This interview schedule will only be used by the Chief Investigator and will not be shared with the participants. The questions have been designed to try and ensure participants are comfortable enough to discuss their beliefs and experiences around shared prescribing decisions in Borderline Personality Disorder. The schedule has been designed as a guide; the interview schedule may evolve during data collection to ensure it adequately reflects issues raised during the preceding interviews. Not all follow up prompt points will be asked to all participants.

**Introduction:**The interview will commence with personal introductions and thanking the participant for their willingness to participate in the research.

**Interview Process:**The Chief Investigator will explain the intended duration of the interview, reiterate the topic, and confirm the process for signalling that an individual wishes to pause or stop the interview.

**Consent:**The Chief Investigator will confirm that the participants’ information leaflet has been read and understood and any remaining questions will be answered.

Consent to participate in the interview, lasting up to one hour, and approval to audio record and take field notes will be obtained from participants, by the Chief Investigator, beforehand and reconfirmed immediately prior to the interview.

Confidentiality, anonymity of data and the right to withdraw from the study at any time will be reiterated.

This consent will be recorded and documented.

**Dissemination of the Research Findings:**Participants will be asked if they wish to be kept informed of the research findings.

**Basic Demographic Information:**
Participants will be asked to self-report the following information at the start of the interview:

- Ethnicity
- Age
- Sex
- Geographical location
- Patient/Carer status

**INTERVIEW**

1. Could you please tell me your experiences of living with, or caring for an individual living with, BPD?

- What do you understand by the term BPD?

2. Could you tell me your experiences about the use of medication for BPD?

- How do you feel generally about the use of medication for BPD?
- What has helped and why?
- What has not helped and why?
- How long was the medication prescribed for?
- What led to that particular drug being prescribed at that particular time?
- What was the goal for this prescription?

3. What do you think influences what is prescribed to you, or the person you care for?

- Planned or unplanned?
- Do you discuss options?
- Was there a goal in starting medication?

4. How much influence do you think that you have on the prescription process?

- Is it a shared decision?
- Why do you think that you have that level of influence?
- If patient, then maybe – do you think that the family carer has much influence? Why?

5. What has and would influence your choices around medication?

- Explanation from healthcare professionals
- Literature resources such as leaflets websites etc
- Diagnosis
- Barriers such as existing preconceptions about services, medication etc.

6. Is there anything else you would like to add to your answers?

**Closing**

Thank you for taking part in this study. Your interview has been audio-recorded and will be transcribed and analysed. If you have opted to receive a summary, we will contact you using the email address provided.
