# Supplementary material for: Patients’ and informal carers’ insights into influences on prescribing in borderline personality disorder: a qualitative interview study in the UK
Source: BMJ Open. 2025 Dec 3;15(12):e108927. doi: 10.1136/bmjopen-2025-108927 (PMC12682173; doi:10.1136/bmjopen-2025-108927)
Supplement: Supplementary file 3 [file bmjopen-2025-108927.reviewer_comments.pdf]

PEER REVIEW HISTORY

BMJ Open publishes all reviews undertaken for accepted manuscripts. Reviewers are asked to complete a checklist review form and are provided with free text boxes to elaborate on their assessment. These free text comments are reproduced below.

ARTICLE DETAILS

Title (Provisional)

Patients’ and Informal Carers’ Insights into Influences on Prescribing in Borderline Personality Disorder: A Qualitative Interview Study in the United Kingdom

Authors

confue, joshua; Maidment, Ian; Jones, Matthew

VERSION 1 - REVIEW

**Reviewer**	**1**
**Name**	**Broadbear, Jillian**
**Affiliation**	**Monash University**
**Date**	**27-Aug-2025**
**COI**	**None**

Please see attachment

British Medical Journal

Patients' and Informal Carers' Insights into Influences on Prescribing in Borderline Personality Disorder: A Qualitative Interview Study

General comments:

This is an interesting study that proposes exploring patient-related factors that influence shared prescribing decisions in the treatment of BPD. It is clearly written and an important topic to explore, given the high prevalence of (poly)pharmacy in people with BPD despite the paucity of evidence or indications for psychoactive medications in its treatment.

A striking omission appears to be the lack of outcomes reported for the presence/absence of collaborative decision making around prescribing (despite use of the word ‘shared’ in the title). Patients report educating themselves (with sources varying in quality), rather than reflecting on discussions they had with their prescriber or pharmacist. Thus, the implied purpose of the study doesn’t seem to be realised as conducted (based on the reporting of results). Moreover, the perspectives of patients

and carers aren't clearly delineated with respect to their contributions to the decision-making process. It is possible that the title and rationale for the study need to be reworded to align with what is being reported.

As an aside, did any participant nominate an occasion when they had declined a prescription based on perceived risks outweighing benefits?

Specific comments:

Abstract:

The Objectives mention 'patient-related factors', however informal carers were also part of the study. This needs to be clarified.

Design – There seems to be a misunderstanding here. A qualitative methodology is not a product of its exploratory nature. It is a specifically chosen methodology that addresses the study aims. Please consider amending.

Strengths and Limitations:

Generally, with qualitative studies, the number of participants is determined by the richness of the data and 'saturation' of themes (lack of novel information with further interviews).

Introduction:

Page 6, Line 14: The prevalence of BPD is in the range of 0.7-1.5%. The number you have cited sounds like an outlier. The lower prevalence makes their representation in services even more extraordinary.

The content of paragraph 3 is a bit confusing. "Notably, despite the significant role of the patient in influencing BPD prescribing decisions…" It is not clear what this expectation is based on. Is this an 'ideal world' recommendation or based on some evidence? If the latter, why is there so little evidence that this happens in practice?

Line 52: Suggestion to add 'reported to "affective changes reported by patients"

Line 54: Please clarify what you mean by "…despite the powerful impact of medication being a major theme."

I don't think that the purpose of the study has been fully articulated. Why is it important that prescribing decisions be collaborative? Is this a particular problem with psychoactive prescribing relative to others, and for people with BPD in particular? Despite the lack of evidence/indications for medications in the treatment for BPD, why is it so frequently a feature of their treatment? How might this kind of prescribing be helpful/harmful, thus requiring informed and collaborative decision making? What is an 'informal carer' and why is it important that they be included in prescribing decisions?

Materials & Methods:

Page 8, line 19: Living with BPD?

Lines 23-4: This sentence seems to be incomplete: "…notably questions and power dynamics in decision-making" Are you referring to their shaping questions asked about power dynamics, or did their presence change the power dynamics in the research team's decision making?

Line 44: Does 'a favourable opinion' mean ethical approval for the study? Consider rewording this.

Page 9, lines 15-18: Were each of these people consulted prior to study inclusion? "…they, the research team, their community psychiatric nurse or responsible clinician considered that involvement could pose them a significant risk of harm." Were any prospective participants excluded on this basis?

Lines 20+: I'm not familiar with setting a target sample size for qualitative studies. I don't think that is necessary here (and sources weren't cited to justify the number chosen). The remaining sentences in this paragraph are sufficient.

Line 32-3: Were participants screened concerning the inclusion criteria prior to study inclusion? I'm unsure why recruiting through social media would have presented a problem in this regard. Was payment offered for participation?

Line 36: Did participants provide written informed consent?

Results:

Page 12: It would assist readability if participants could be identified as 'patient' or 'carer' in the quotations.

Page 13: Please clarify "In both participants who wanted to start and stop medication,…"

There is little evidence of collaborative decision-making in the way that the results are presented. Was this asked directly of participants?

Page 14, Line 43: Please define 'EUPD'; perhaps just replace with '[BPD]'?

Lines 54-56: The purpose of including this quotation isn't clear. If this saying that symptoms common to other mental health conditions weren't being treated with medication? This seems unlikely given the polypharmacy that often occurs with treating BPD (including antidepressants, anxiolytics, ECT etc.)

The contributions of patients are carers aren't clearly delineated – did carers have different perspectives?

Discussion:

The short, disjointed paragraphs could be synthesized according to theme to build a more detailed argument/explanation for each of the findings.

The quality of information that patients access to guide their decisions needs to be discussed. Can they be considered informed decision-makers given the sources they describe? What about (possibly the absence of) information supplied by the prescriber during the prescribing decisions?

What was learned from the contributions of informal carers?

Was data saturation reached in terms of contributions to the thematic analysis? Commenting on this would be more relevant that explaining why more participants could not be recruited. Qualitative research does not generally require large numbers of participants.

It is better to state ‘paper in preparation’ rather than PAPER 2.

Final paragraph, page 16: This is a good opening to discuss the quality of information that patients access to base their opinions on. In an ideal world, what is the role of the prescriber – and perhaps the pharmacist – in this process?

Page 17, 2nd paragraph: It is unclear why this paragraph has been included, as the Results did not refer to which medication classes were being prescribed.

Paragraph 3: The primary interpersonal interactions that participants referred to were peer reports on social media. This is worth exploring.

Paragraph 4: This doesn’t seem to fit, given the present paper doesn’t ask about or describe results about the therapeutic relationship. Indeed, this seems an omission, along with the lack of results concerning shared decision-making. HCP is defined here (for the first time?) This paragraph is quite confusing, as it doesn’t seem to be referring to the results as presented.

Paragraph 5: This seems to align with the earlier mention of patient expectations/placebo effect. Consider combining these into a single paragraph.

Page 18, first paragraph: This is a good point. Also important is the real/perceived lack of other treatment options (e.g. as described in the 3rd paragraph).

Line 11: Please clarify “even prior adverse effects”

2nd paragraph: Could the discounting of potential side effects also be due to the quality of information available to participants?

Line 39: Please reword “or a crisis lines”

Page 20, second paragraph: What do you think was the purpose of the stipulation that participants share the contact details of their prescriber? Was this a safety feature?

Was their HCP notified of their involvement in this study? If so, that might constitute a violation of the patient's autonomy. If it was a safety step – with HCP only contacted in the event of a harmful outcome on the patient as a result of study participation – this seems quite a usual precaution. Also, it's unusual to 'blame' the ethics process as a limitation of the study, given the priority of the ethics process to safeguard the wellbeing of research participants.

An important finding (if this is what the study was investigating) is the lack of collaborative decision making in prescribing for BPD.

With respect to the CONSORT criteria,

#7 What did the participant know about the researcher? Was this described in the information and consent form?

#22 Data saturation was in fact mentioned in the manuscript

**Reviewer**	**2**
**Name**	**Theodoros, Theo**
**Affiliation**	**Belmont Private Hospital, Psychiatry**
**Date**	**01-Sep-2025**
**COI**	**None**

As a practicing psychiatrist in Australia who is also a registered pharmacist, I'm very impressed with this research and very much want to commend the authors. They have very much taken a patient centred approach which is what I do in my clinical practice, and their findings are very much in alignment with my clinical experience. I lament that it's unfortunate that the researchers didn't have psychiatrists included in the research, but this is likely reflective of the academic divide between the disciplines of psychiatry and pharmacy that we also see in Australia. .

More reflection and data on the type of psychotropic medication would have been useful. Irrespective, I find this very useful research and I commend the authors for their efforts. If they would benefit from collaborating with a psychiatrist in future similar research, I'm very willing to support, as this is vital research for mental health, and crucial for multi-disciplinary research to flourish.

VERSION 1 - AUTHOR RESPONSE

Reviewer: 1

1. **This is an interesting study that proposes exploring patient-related factors that influence shared prescribing decisions in the treatment of BPD. It is clearly written and an important topic to explore, given the high prevalence**

of (poly)pharmacy in people with BPD despite the paucity of evidence or indications for psychoactive medications in its treatment.

We thank the reviewer for their thoughtful summary and recognition of the importance of this topic.

2. **A striking omission appears to be the lack of outcomes reported for the presence/absence of collaborative decision making around prescribing (despite use of the word 'shared' in the title).**

   Patients report educating themselves (with sources varying in quality), rather than reflecting on discussions they had with their prescriber or pharmacist. Thus, the implied purpose of the study doesn't seem to be realised as conducted (based on the reporting of results).

We thank the reviewer for this valuable observation. To clarify, our use of the term shared in the title was intended to denote that treatment decisions ideally occur within the framework of shared decision-making . However, the study was not designed to evaluate the extent or outcomes of shared decision-making . Instead, our aim was to explore the influences that drove prescribing decision, through the exploration of the lived experiences of patients' and informal carers
We have revised the title

"Patients' and Informal Carers' Insights into Influences on Prescribing in Borderline Personality Disorder: A Qualitative Interview Study in the United Kingdom"

We have also clarified this distinction in the Introduction and Discussion to avoid potential misinterpretation for example on Page 6, Line 139-143:

"Given the limited efficacy of medications in BPD, the high rates of prescribing, there is a need to better understand the factors driving these practices. This study seeks to expand the limited existing evidence base by exploring the influences shaping prescribing decisions from both patient and carer perspectives to better inform our understanding of the factors behind prescribing in BPD."

3. **Moreover, the perspectives of patients and carers aren't clearly delineated with respect to their contributions to the decision making process.**

We thank the reviewer for raising this important point. On reviewing the data, we found that the perspectives of patients and carers were highly aligned. While carers sometimes provided additional context regarding safety and monitoring, these themes were also echoed by patients, and there was no consistent divergence in focus between the two groups. We have clarified this in the Discussion sections to make explicit that the contributions of patients and carers were aligned on Page 14 , Line 381-384:

"These themes were evident across the accounts of both patients and informal carers. While carers provided additional insights, particularly in relation to risk, monitoring, and

support needs, the perspectives they offered were largely consistent with those expressed by patients."

4. **It is possible that the title and rationale for the study need to be reworded to align with what is being reported.**

We thank the reviewer for this valuable observation. In response, we have revised the title and refined the rationale to ensure that they accurately reflect the study's focus on patient-related factors influencing prescribing decisions. We have now removed the term shared prescribing from the objectives to avoid ambiguity. In addition, we have clarified in the Introduction and Methods sections that the reference to shared decision making was intended to highlight best practice in prescribing, and to explain the rationale for exploring patient perspectives Page 5, Line 120-122:

"Modern prescribing is ideally based around the shared decision-making process, with the decision to prescribe made jointly between the prescriber and the patient or their representative"

5. **"Notably, despite international advocacy for patient-centred care and active patient involvement in prescribing decisions..."**

   As an aside, did any participant nominate an occasion when they had declined a prescription based on perceived risks outweighing benefits

We thank the reviewer for their query. Yes, participants described instances where they had either declined a prescription or ceased taking a medication because they perceived the risks to outweigh the benefits.

Abstract

6. **The Objectives mention 'patient-related factors', however informal carers were also part of the study. This needs to be clarified.**

We thank the reviewer for highlighting this important point. We have revised the Objectives to clarify that the study explored factors influencing prescribing decisions from both patient and informal carer perspectives Page 3 , Line 62-63:

"To explore the patient- and informal-carer reported factors that influence shared prescribing decisions in the management of borderline personality disorder (BPD)."

7. **Design – There seems to be a misunderstanding here. A qualitative methodology is not a product of its exploratory nature. It is a specifically chosen methodology that addresses the study aims. Please consider amending.**

We appreciate this clarification. We have amended the description of the study design to explicitly state that a qualitative methodology was purposefully chosen to address the study aims, rather than implying it arose from the exploratory nature of the research Page 3, Line 65-67:

"The study employed a qualitative methodology, of semi-structured interviews with patients and informal carers to examine perspectives on prescribing decisions and the factors shaping them"

8. **Strengths and Limitations: Generally, with qualitative studies, the number of participants is determined by the richness of the data and 'saturation' of themes (lack of novel information with further interviews).**

Thank you for this observation. We have removed the line.

<u>**Introduction:**</u>

9. **Page 6, Line 14: The prevalence of BPD is in the range of 0.7-1.5%. The number you have cited sounds like an outlier. The lower prevalence makes their representation in services even more extraordinary.**

We thank the reviewer for drawing attention to this important point. The previously quoted prevalence figure was from primary care data. We have now revised the statement to clarify that epidemiological studies report prevalence rates ranging from 0.7% to 2.7%. on Page 5, Line 106-108**:**

"Estimates of the lifetime prevalence of BPD vary across studies, ranging from approximately 0.7% to 2.7% in the general population "

10. **The content of paragraph 3 is a bit confusing. "Notably, despite the significant role of the patient in influencing BPD prescribing decisions…" It is not clear what this expectation is based on. Is this an 'ideal world' recommendation or based on some evidence? If the latter, why is there so little evidence that this happens in practice?**

We thank the reviewer for this thoughtful observation. We agree that the phrasing could be clearer. Our intention was to highlight that guidelines and shared decision-making frameworks emphasise the patient's role in influencing prescribing decisions and have revised the text to clarify this on Page 5 , Line 125-126**:**

"Notably, despite international advocacy for patient-centred care and active patient involvement in prescribing decisions, only two papers…"

11. **Line 52: Suggestion to add 'reported to "affective changes reported by patients"**

We thank the reviewer for this helpful suggestion. We have revised the text accordingly to read on Page 5 , Line 134-135**:**

"affective changes reported by patients"

12. **Line 54: Please clarify what you mean by "…despite the powerful impact of medication being a major theme."**

We appreciate this request for clarification from the reviewer. Our intention was to convey the conflict within the previous literature that patients often described the substantial impact of medication , alongside uncertainty about the effect of medication. We have revised the section to provide clarify on Page 5, Line 135-137:

"Contrastingly, Patel (2020), reported that patients often expressed uncertainty about the effects of medication, while paradoxically also frequently describing its powerful impact."

13. **I don't think that the purpose of the study has been fully articulated.**
    Why is it important that prescribing decisions be collaborative?
    Is this a particular problem with psychoactive prescribing relative to others, and for people with BPD in particular?
    Despite the lack of evidence/indications for medications in the treatment for BPD, why is it so frequently a feature of their treatment?
    How might this kind of prescribing be helpful/harmful, thus requiring informed and collaborative decision making?

We thank the reviewer for this valuable observation. To clarify, the objective of this study was to explore, from the perspective of patients and informal carers, why despite the limited evidence and indications for medication use in BPD, such prescribing remains common. We did this by exploring their lived experience to identify potential factors that influence prescribing decisions. We have amened the objective and introduction to clarify this Page 3 , Line 62-68:

"To explore the patient and informal-carer reported factors that influence prescribing decisions in the management of borderline personality disorder (BPD)."

The term "shared decision" was initially introduced because, at the early stages of the project, we were often asked why we planned to interview patients about prescribing decisions. We have revised the paper , by removing "shared" to clarify this throughout the manuscript

We have also amened the end of the introduction to better articulate the purpose of this study and the rational Page 6 , Line 139-143:

"Given the limited efficacy of medications in BPD, the high rates of prescribing, there is a need to better understand the factors driving these practices. This study seeks to expand the limited existing evidence base by exploring the influences shaping

prescribing decisions from both patient and carer perspectives to better inform our understanding of the factors behind prescribing in BPD. "

14. **What is an 'informal carer' and why is it important that they be included in prescribing decisions?**

We thank the reviewer for this comment. We feel the inclusions of informal carers reflects the collaborative nature of healthcare, where treatment decisions are often influenced by those providing informal support. This is particularly poignant in mental health patients, where there are more likely to be times patients are detained against there will, or lack capacity to make decisions themselves.
We have also defined the term informal carer as a family member, partner, or friend providing unpaid support , Page 6, Line 147-148**:**

"Informal carers being defined as a family member, partner, or friend providing unpaid support."

Materials & Methods:

15. **Page 8, line 19: Living with BPD?**

We thank the reviewer for this suggestion and amended the manuscript on Page 6, Line 155:

"Four participants involved in treating or living with BPD"

16. **Lines 23-4: This sentence seems to be incomplete: "...notably questions and power dynamics in decision-making" Are you referring to their shaping questions asked about power dynamics, or did their presence change the power dynamics in the research team's decision making?**

We thank the reviewer for this comment. We have clarified in the Method that the stakeholder input was key in highlighting relational issues as a factor in prescribing decisions on Page 6, Line 157-158**:**

"Their input supported the development of the protocol and related materials, particularly by highlighting the importance of relational factors in decision-making."

17. **Line 44: Does 'a favourable opinion' mean ethical approval for the study? Consider rewording this.**

We thank the reviewer for this comment, 'Favourable opinion' is the official term used for approval in the UK. For clarity, we have amended the wording to approval on Page 6, Line 171**:**

18. **Page 9, lines 15-18: Were each of these people consulted prior to study inclusion? "…they, the research team, their community psychiatric nurse or responsible clinician considered that involvement could pose them a significant risk of harm." Were any prospective participants excluded on this basis?**

We thank the reviewer for raising this point, and we have clarified this section, on Page 8, Line 190-193:

"Participants were excluded if they, the research team, or their community psychiatric nurse or responsible clinician (for those referred by healthcare professionals) believed that participation posed a significant risk of harm."

We can confirm that no prospective participants were excluded through this screening process, and that the screening process was approved by a independent ethics board.

19. **Lines 20+: I'm not familiar with setting a target sample size for qualitative studies. I don't think that is necessary here (and sources weren't cited to justify the number chosen). The remaining sentences in this paragraph are sufficient.**

We thank the reviewer for raising this important point, and agree that the target was potentially confusing. Our reference to an anticipated number of participants was not intended as a formal sample size but as a estimate informed by qualitative research and our prior estimation of the sample required to generate adequate insight based on the principle of information power.

This approach still recognises that the adequacy of the final sample depends on the richness and relevance of the data in addressing the research question.
To clarity, we have revised manuscript on Page 8, Line 194-198:

"Recruitment was guided by the principle of information power, with an initial recruitment target of up to 20 participants being set. This estimate was based on existing qualitative research and our prior estimation of the sample size required to generate adequate insight. Yet , the final sample determined by the richness and sufficiency of the data to address the study aims(16).

20. **Line 32-3: Were participants screened concerning the inclusion criteria prior to study inclusion? I'm unsure why recruiting through social media would have presented a problem in this regard. Was payment offered for participation?**

We thank the reviewer for raising this point. Recently in the UK, researchers have found that social recruitment media recruitment for studies offering vouchers and with self-reported screen against inclusion criteria has led to the recruitment of participants who are likely not to actually meet the inclusion criteria. On the advice of our university, we

therefore chose not to use social media. We have clarified that reimbursement was offered in the form of a voucher on Page 8, Line 208-209**:**

“All participants were offered a £30 voucher for their participation.”

21. **Line 36: Did participants provide written informed consent?**

We thank the reviewer for highlighting this omission we have added clarification on Page 8, Line 207-208**:**

“After which written consent was obtained prior to conducting all interviews.”

Results:

22. **Page 12: It would assist readability if participants could be identified as ‘patient’ or ‘carer’ in the quotations.**

We thank the reviewer for this helpful suggestion. We have now identified whether quotations come from patients or carers, which improves both clarity and readability.

23. **Page 13: Please clarify “In both participants who wanted to start and stop medication,…” There is little evidence of collaborative decision-making in the way that the results are presented. Was this asked directly of participants?**

We appreciate this important point from the reviewer. We have revised the sentence for clarity on Page12, Line 287-289**:**

“Among participants who described wanting to start or stop medication, there seemed to be a greater belief in self identified resources, internet forums and social media , rather than the information provided by HCPs.”

We confirm that collaborative decision-making was explored directly through the interview schedule as a prompt See Appendix B

24. **Page 14, Line 43: Please define ‘EUPD’; perhaps just replace with ‘[BPD]’?**

We thank the reviewer for this observation. To avoid confusion, we have replaced ‘EUPD’ with ‘BPD’, in this instance on Page 13, Line 341**:**

25. **Lines 54-56: The purpose of including this quotation isn’t clear. If this saying that symptoms common to other mental health conditions weren’t being treated with medication? This seems unlikely given the polypharmacy that often occurs with treating BPD (including antidepressants, anxiolytics, ECT etc.)**

We are grateful for this comment from the reviewer. The quotation illustrates how participants perceived that they were unable to access services such as CBT, that was available for those with other diagnosis like depression. Page 13, Line 344-345**:**

"Even where services were seen positively, a consistent issue was the slow pace of, or lack of access to, a particular type of treatment (often psychological therapy): ...

Page 13, Line 342-344**:**

"My personal experience: there's no difference with depressive, depressive lows. We get anxiety; we get all of those broad spectrum of emotions, very intensely and so why that help isn't available" Participant-002 (Patient)"

26. **The contributions of patients are carers aren't clearly delineated – did carers have different perspectives?**

We thank the reviewer for raising this important point. While analysing the data, we found that the perspectives of patients and carers were highly aligned, we have revised the manuscript to make this explicit on Page 15, Line 381-384**:**

"These themes were evident across the accounts of both patients and informal carers. While carers occasionally provided additional insights, particularly in relation to risk, monitoring, and support needs, the perspectives they offered were largely consistent with those expressed by patients. ."

Discussion:

27. **The short, disjointed paragraphs could be synthesized according to theme to build a more detailed argument/explanation for each of the findings.**

We appreciate this feedback from the reviewer, we have restructured the Discussion by introducing subheadings, alongside a general review to provide a more coherent narrative.

28. **The quality of information that patients access to guide their decisions needs to be discussed. Can they be considered informed decision-makers given the sources they describe? What about (possibly the absence of) information supplied by the prescriber during the prescribing decisions?**

We thank the reviewer for this important suggestion. We have expanded the Discussion to critically consider the quality and sources of information available to patients. We highlight the reliance on peers and online sources, and the potential limitation of such on Page 16, Line 4423-426**:**

", and therefore placebo responses, are often shaped by the sources of information they rely on. Many participants placed greater weight on the lived experiences of others

with the same condition than on HCPs. Social media was Patients' expectations particularly prominent..."

We have also include a comment about the effect of the information utilised on the prescriber process on Page 16, Line 431-432

"This raises important questions about the extent to which patients can be regarded as informed decision-makers, and about the influence of preconceived expectations on treatment outcomes."

29. **What was learned from the contributions of informal carers?**

We thank the reviewer for this comment and have added a section to explore the contribution of patients and informal carers on Page 15, Line 381-384**:**

"These themes were evident across the accounts of both patients and informal carers. While carers provided additional insights, particularly in relation to risk, monitoring, and support needs, the perspectives they offered were largely consistent with those expressed by patients"

30. **Was data saturation reached in terms of contributions to the thematic analysis? Commenting on this would be more relevant that explaining why more participants could not be recruited. Qualitative research does not generally require large numbers of participants.**

We thank the reviewer for this point though we opted to utilise the concept of information power. While overlapping with data saturation, information power places greater emphasis on initial sampling and the extent to which the data meaningfully contributes to addressing the research question.

That being said we feel that data collected was of sufficient quality to make such as contribution we have clarified this on

Page 15, Line 401-403**:**

"... , the data collected was of sufficient quality and relevance to address the study aims and to make a meaningful contribution to the field."

31. **It is better to state 'paper in preparation' rather than PAPER 2.**

We have amended the text in line with the reviewer's recommendations throughout the manuscript for example on Page 154, Line 408.

32. **Final paragraph, page 16: This is a good opening to discuss the quality of information that patients access to base their opinions on. In an ideal world, what is the role of the prescriber – and perhaps the pharmacist – in this process?**

We thank the reviewer for this helpful suggestion. We have extended this section to discuss the potential roles of prescribers and pharmacists in supporting informed patient decision Page 16, Line 421-422**:**

"highlighting the crucial role of healthcare professionals in supporting individuals to make informed decisions about their medications."

33. **Page 17, 2nd paragraph: It is unclear why this paragraph has been included, as the Results did not refer to which medication classes were being prescribed**.

We appreciate this observation from the reviewer and have removed the paragraph for clarity.

34. **Paragraph 3: The primary interpersonal interactions that participants referred to were peer reports on social media. This is worth exploring.**

We thank the reviewer for highlighting this point. We have expanded our Discussion regarding this interaction on Page 16, Line 424-425**:**

"Many participants placed greater weight on the lived experiences of others with the same condition, than on HCPs. "

Page 15, Line 427-429**:**

"A 2024 study found that this community may be a more appealing source of information with social media offering a distraction from interpersonal problems; a reassurance that people are still cared for; an outlet for those struggling with self-esteem ..."

35. **Paragraph 4: This doesn't seem to fit, given the present paper doesn't ask about or describe results about the therapeutic relationship. Indeed, this seems an omission, along with the lack of results concerning shared decision-making. HCP is defined here (for the first time?) This paragraph is quite confusing, as it doesn't seem to be referring to the results as presented.**

We thank the reviewer for this helpful observation. We agree that our original wording was unclear. While our dataset contained little direct material on the influence of the therapeutic relationship on decision-making in prescribing, we sought to situate participants' positive beliefs about medication within the broader literature. We have now revised this paragraph to improve clarity and to ensure it aligns more closely with the results we present on Page 16, Line 434-444:

"With regard to the validating effect of medication. The act of prescribing can provide benefits beyond the pharmacological, it can provide secondary benefits ... prescribing as a means of validation ...has previously been reported by healthcare professionals as

a driver of prescribing ... the importance of the therapeutic relationship did not emerge as a prominent theme in prescribing within this study..."

The terms HCP as Healthcare Professional HCP is defined in the introduction on Page5 Line 124.

36. **Paragraph 5: This seems to align with the earlier mention of patient expectations/placebo effect. Consider combining these into a single paragraph**.

We thank the reviewer for this suggestion. We have combined these sections on Page 16, Line 423-433**.**

37. **Page 18, first paragraph: This is a good point. Also important is the real/perceived lack of other treatment options (e.g. as described in the 3rd paragraph).**

We thank the reviewer for this positive feedback.

38. **Line 11: Please clarify "even prior adverse effects"**

We thank the reviewer for this comment. We have revised the sentence to clarify that it refers specifically to concerns about potential adverse effects, including prior adverse effects experienced by participants with other medications on Page 17, Line 462-463**:**

"Concerns around potential adverse effects, and even prior adverse effects experienced by participants from other medications , were also reported as influencing decisions ..."

39. **2nd paragraph: Could the discounting of potential side effects also be due to the quality of information available to participants?**

We thank the reviewer for this suggestion. We have incorporated this perspective into the Discussion, noting that the minimisation of potential side effects may be linked to limited or poor-quality information sources Page 17, Line 471-473**:**

"This may be because fear of side effects can differ based on personal and clinical attributes, as well as the quality of information individuals utilise, among other factors"

40. **Line 39: Please reword "or a crisis lines"**

We have reworded this on Page 17, Line 483**:**

"mental health crisis service "

41. **Page 20, second paragraph: What do you think was the purpose of the stipulation that participants share the contact details of their prescriber? Was this a safety feature? Was their HCP notified of their involvement in this study? If so, that might constitute a violation of the patient's autonomy. If it was a safety step – with HCP only contacted in the event of a harmful**

outcome on the patient as a result of study participation – this seems quite a usual precaution. Also, it's unusual to 'blame' the ethics process as a limitation of the study, given the priority of the ethics process to safeguard the wellbeing of research participants.

We thank the reviewer for this thoughtful comment. The stipulation that participants provide their prescriber's contact details was an ethics committee requirement, intended solely as a safety precaution. Importantly, prescribers were not notified of participants' involvement in the study unless a serious concern for participant safety arose, which did not occur.

We acknowledge, however, that this requirement posed challenges for recruitment and engagement. Several participants expressed that being asked to provide prescriber details before being able to share their experiences felt undermining of their autonomy and, in some cases, reinforced stigma. We note that similar qualitative studies involving people with severe mental illness have not always required this measure and rarely for physical health studies, suggesting some variability in how committees interpret and apply safety safeguards.

In light of this, we have revised the manuscript to clarify that our intention was not to "blame" the ethics process, but to highlight how this stipulation while designed to protect participants may also have unintentionally introduced barriers to participation on Page 15, Line 404-407**:**

"This lack of trust may have been reinforced by the requirement for the researchers to have contact details for the HCP or support network of each participant."

42. **An important finding (if this is what the study was investigating) is the lack of collaborative decision making in prescribing for BPD.**

We thank the reviewer for this comment and have clarified throughout the manuscript that we aimed to explore the influences that drove prescribing decisions.

43. **CONSORT criteria**

- #7 What did the participant know about the researcher? Was this described in the information and consent form?

We thank the reviewer for highlighting this aspect of the Consort form. The participant information leaflet included details of the researcher, including their name, their organisation, supervisor and purpose of the research. We have added this explicitly to the consort form in Appendix B

- #22 Data saturation was in fact mentioned in the manuscript

We thank the reviewer for this helpful observation. We recognise that while the manuscript focused on the concept of information power in determining sample adequacy , there is conceptual overlap with data saturation. To clarify this relationship,

we have revised Appendix B to explicitly acknowledge this overlap and to explain that our approach was informed by information power.

Reviewer: 2

1. **As a practicing psychiatrist in Australia who is also a registered pharmacist, I'm very impressed with this research and very much want to commend the authors. They have very much taken a patient centred approach which is what I do in my clinical practice, and their findings are very much in alignment with my clinical experience.**

We are very grateful for the reviewers positive feedback. We appreciate your recognition of the patient-centred approach and value of this work.

2. **I lament that it's unfortunate that the researchers didn't have psychiatrists included in the research, but this is likely reflective of the academic divide between the disciplines of psychiatry and pharmacy that we also see in Australia.**

We thank the reviewer for highlighting this important point. We agree that including psychiatrists could have added diversity to our research. While we did have a psychiatrist as a stakeholder in the development of the this study, the current study reflects some of the disciplinary boundaries that exist within academic and clinical contexts. We recognise this as a an opportunity for strengthening future work. Moving forward, we would be keen to collaborate with psychiatrists both in the UK and aboard (and please feel free to email members of the team to discuss collaboration including [i.maidment@aston.ac.uk; jc517@bath.ac.uk] once the paper review has been completed

3. **More reflection and data on the type of psychotropic medication would have been useful.**

We thank the reviewer for this thoughtful comment. We agree that the type of psychotropic medication prescribed can provide important contextual information. In response, we have expanded the introduction on the type of medications often prescribed in this cohort on Page 5, Line 116-118:

"Commonly prescribed medications include antidepressants, mood stabilisers, antipsychotics, and anxiolytics, which are often administered over extended periods of time (8)."

4. **Irrespective, I find this very useful research and I commend the authors for their efforts. If they would benefit from collaborating with a psychiatrist in future similar research, I'm very willing to support, as this is vital research for mental health, and crucial for multi-disciplinary research to flourish.**

We sincerely thank the reviewer for the generous offer of collaboration and your recognition of the importance of multi-disciplinary research in this field. We strongly agree that such collaboration is important to enhance understanding and improve patient care.

These revisions, along with a comprehensive review and expansion of the manuscript, have significantly enhanced the coherence, depth, and impact of our manuscript. We believe that the revised manuscript effectively addresses the reviewers' concerns and is now well-suited for publication in British Medical Journal Open.
